# *Rheumatic*?—A Digital Diagnostic Decision Support Tool for Individuals Suspecting Rheumatic Diseases: A Multicenter Pilot Validation Study

**DOI:** 10.3389/fmed.2022.774945

**Published:** 2022-04-25

**Authors:** Rachel Knevel, Johannes Knitza, Aase Hensvold, Alexandra Circiumaru, Tor Bruce, Sebastian Evans, Tjardo Maarseveen, Marc Maurits, Liesbeth Beaart-van de Voorde, David Simon, Arnd Kleyer, Martina Johannesson, Georg Schett, Tom Huizinga, Sofia Svanteson, Alexandra Lindfors, Lars Klareskog, Anca Catrina

**Affiliations:** ^1^Leiden University Medical Center, Leiden, Netherlands; ^2^Translational and Clinical Research Institute, Newcastle University, Newcastle upon Tyne, United Kingdom; ^3^Department of Internal Medicine 3, Friedrich-Alexander-Universität Erlangen-Nürnberg and Universitätsklinikum Erlangen, Erlangen, Germany; ^4^Deutsches Zentrum für Immuntherapie, Friedrich-Alexander-Universität Erlangen-Nürnberg and Universitätsklinikum Erlangen, Erlangen, Germany; ^5^Université Grenoble Alpe, Autonomie, Gérontologie, E-santé, Imagerie et Société, Grenoble, France; ^6^Division of Rheumatology, Department of Medicine, Karolinska University Hospital, Karolinska Institutet, Stockholm, Sweden; ^7^Center for Rheumatology, Academic Specialist Center, Stockholm, Sweden; ^8^Ocean Observations AB, Design Consultancy, Stockholm, Sweden; ^9^Elsa Science AB, Digital Health Company, Stockholm, Sweden; ^10^Master Advanced Nursing Practice, University of Applied Sciences Leiden, Leiden, Netherlands

**Keywords:** eHealth, symptom assessment, diagnostics, rheumatology, mHealth (mobile health)

## Abstract

**Introduction:**

Digital diagnostic decision support tools promise to accelerate diagnosis and increase health care efficiency in rheumatology. *Rheumatic?* is an online tool developed by specialists in rheumatology and general medicine together with patients and patient organizations. It calculates a risk score for several rheumatic diseases. We ran a pilot study retrospectively testing *Rheumatic?* for its ability to differentiate symptoms from existing or emerging immune-mediated rheumatic diseases from other rheumatic and musculoskeletal complaints and disorders in patients visiting rheumatology clinics.

**Materials and Methods:**

The performance of *Rheumatic?* was tested using in three university rheumatology centers: (A) patients at Risk for RA (Karolinska Institutet, *n* = 50 individuals with musculoskeletal complaints and anti-citrullinated protein antibody positivity) (B) patients with early joint swelling [dataset B (Erlangen) *n* = 52]. (C) Patients with early arthritis where the clinician considered it likely to be of auto-immune origin [dataset C (Leiden) *n* = 73]. In dataset A we tested whether *Rheumatic?* could predict the development of arthritis. In dataset B and C we tested whether *Rheumatic?* could predict the development of an immune-mediated rheumatic diseases. We examined the discriminative power of the total score with the Wilcoxon rank test and the area-under-the-receiver-operating-characteristic curve (AUC-ROC). Next, we calculated the test characteristics for these patients passing the first or second expert-based *Rheumatic?* scoring threshold.

**Results:**

The total test scores differentiated between: (A) Individuals developing arthritis or not, median 245 vs. 163, *P* < 0.0001, AUC-ROC = 75.3; (B) patients with an immune-mediated arthritic disease or not median 191 vs. 107, *P* < 0.0001, AUC-ROC = 79.0; but less patients with an immune-mediated arthritic disease or not amongst those where the clinician already considered an immune mediated disease most likely (median 262 vs. 212, *P* < 0.0001, AUC-ROC = 53.6). Threshold-1 (advising to visit primary care doctor) was highly specific in dataset A and B (0.72, 0.87, and 0.23, respectively) and sensitive (0.67, 0.61, and 0.67). Threshold-2 (advising to visit rheumatologic care) was very specific in all three centers but not very sensitive: specificity of 1.0, 0.96, and 0.91, sensitivity 0.05, 0.07, 0.14 in dataset A, B, and C, respectively.

**Conclusion:**

*Rheumatic?* is a web-based patient-centered multilingual diagnostic tool capable of differentiating immune-mediated rheumatic conditions from other musculoskeletal problems. The current scoring system needs to be further optimized.

## Introduction

Despite generally increasing digitalization in rheumatology (1–4), the decision for referral of new patients with suspect rheumatic disease is mostly analog with few exceptions and has not changed in the last decades (5, 6).

Diagnostic delays do not seem to improve significantly (7, 8) and often inhibit early, and therefore effective, therapy. Up to 60% of new referrals to rheumatologists end up having no immune-mediated rheumatic disease (9, 10). In contrast to emergency medicine (11), rheumatology has not yet developed objective and transparent triage standards, further complicating patient referral. Incomplete, illegible and non-importable paper-based referral forms constitute bottlenecks in current clinical care.

Digital diagnostic decision support systems (DDSS) and in particular online self-referral (OSR) systems and symptom checkers (SC) promise to accelerate diagnosis in rheumatic diseases (9, 12–15) and improve health care efficiency in rheumatic diseases. Currently more than 100 SCs exist (16) and are increasingly used by patients (4). Only a minority of these SCs showed transparent, published, promising evidence before being publicly available (17–19). The inclusion of clinical experts and patients in the development process has been recommended by various rheumatology societies (20, 21).

*Rheumatic?* is such a web-based screening tool available in Swedish, English, German and Dutch (15). It is developed by designers, engineers, clinical experts and patients. This screening tool is designed to capture individuals at high risk for developing a rheumatic disease as well as patients with early signs of specific immune-mediated rheumatic diseases. The initial scoring system was constructed by experts in the respective rheumatic diseases included in the screening tool. The aim of this pilot study was to test this multilingual, comprehensive DDSS in individuals suspecting a rheumatic disease to analyze its discriminatory ability between patients with and without immune-mediated rheumatic diseases (imRD).

## Materials and Methods

### *Rheumatic*? A Web-Based Screening Tool

*Rheumatic?* is a web-based screening tool to identify individuals with early signs of, or at high risk for developing Rheumatoid Arthritis, Ankylosing Spondylitis, Systemic Lupus Erythematosus, Myositis, Systemic Sclerosis, or Sjögren’s Syndrome (in this paper called immune-mediated rheumatic diseases; imRD) (15). A team of designers, engineers, clinical experts for the specific diseases, patients, and at risk individuals worked together to build a test that was both medically correct and effortless to use for an average person. The weights and thresholds for scoring *Rheumatic?* were defined based on expert opinion about the relative informative value of the different questions when they are used in the clinical assessment. For this no patient data was used. In short, a first draft of the questions was made, which was then reviewed and adapted in a collaborative workshop with several experts, to make sure the questions covered the most important symptoms of each diagnosis while still keeping it as short and relevant as possible. In this workshop, a first draft of the scoring was made, by having the experts approximate how important each question and option was for each diagnosis relative to the other questions. These scorings were then implemented as an interactive prototype where different combinations (patient vignettes) of answers could be tested, and iteratively improved and revised with input from the experts in several meetings during 2018. Supplementary Table 1 contains the set of questions.

In the next step these experts weighted the importance, the sensitivity and the specificity of each question for a specific disease. The weight of the questions was then used to set threshold-1 for any of the diseases, with the advice to visit a general physician and threshold-2 for any disease, with the advice to visit a rheumatologist (13) (see scores in Supplementary Table 2).

The tool was constructed as part of the JPAST project and versions supported by the EU EIT Health program, and versions in Dutch, English, German and Swedish are at the moment accessible for researchers.

### Study Design

This pilot study utilized retrospective data collection, where research patient records were used to fill out the questionnaire. In this pilot phase, we aimed to test the ability to differentiate between immune-mediated and other rheumatic diseases (osteoarthritis (OA) and gout). The retrospective design allowed us to include a sufficient number of patients with different rheumatic diagnoses.

We also studied three clinical settings covering different stages of disease development. The datasets contained a) at Risk-RA individuals where we tested the ability of Rheumatic? to identify the development of arthritis) patients with early unclassified joint swelling without a clear diagnosis and c) patients with early unclassified joint swelling without a clinical suspicion of an underlying immune mediated disease. In the latter two datasets the different final diagnoses, established after an additional follow-up of these patients for one year, were retrieved from clinical records.

#### Dataset A—Karolinska Institutet

For our analyses we retrospectively selected patients. We selected 50 individuals from Karolinska Risk RA prospective cohort study (22) with at least two years follow up time, in such a way that 42% of the included patients had developed arthritis during follow up period. Patients in this cohort were all referred from non-rheumatology-specialist (in most of the cases primary care doctor) due to suspicions of rheumatic disease. The included patients had musculoskeletal complaints, were ACPA positive and lacked clinical or ultrasound-based arthritis at the first visit at Karolinska University hospital or Center for Rheumatology in Stockholm, Sweden. The main final endpoint was defined by identification of arthritis in at least one joint by clinical examination. Additionally, individuals were identified who had developed RA defined by current classification criteria for RA (23).

#### Dataset B—Erlangen University Medical Centre

Patients referred from a non-rheumatologist-specialist visiting the rheumatology clinic of the Erlangen University Medical Centre with unclassified joint swelling from a prospective cohort study were included. We randomly selected 51 patients with a follow-up of at least one year and a final diagnosis from this cohort. We grouped patients by disease and randomly selected patients from the disease groups, again aiming for at least 50% patients with non-immune-mediated rheumatic disease (osteoarthritis and gout). Final diagnosis was based on clinical records.

#### Dataset C—Leiden University Medical Centre

Patients visiting the rheumatology clinic of the Leiden University Medical Centre with an initial diagnosis of unclassified joint swelling suggestive for synovitis recruited were included in the Leiden Early Arthritis Clinic (24). After one year of follow-up the final diagnosis was registered based on the clinical records. Since this cohort aims to include patient with early immune mediated rheumatic diseases osteoarthritis and gout are less prevalent than in the general outpatient clinic. Given the low prevalence of gout and osteoarthritis here, we aimed for a variety of diagnoses with at least 70% immune-mediated rheumatic disease. We randomly selected 72 patients from this cohort.

### Statistical Analysis

*Rheumatic?* gives a total score, which is built from the individual scores for each of the six diseases. For each disease a participant can pass a first or second threshold (Supplementary Table 2), which aims to inform the participant about the likelihood of having a rheumatic disease. We tested the performance of:

a. the total *Rheumatic?* score using Wilcoxon rank test and the area-under-the-receiver-operating-curve (AUC-ROC).
b. the sensitivity and specificity for having an immune-mediated rheumatic disease when passing threshold 1.
c. the sensitivity and specificity for having an immune-mediated rheumatic disease when passing threshold 2.

All analyses were performed using R. AUC-ROC were calculated using the *pROC* package.

## Results

### Patient Characteristics

By design, each setting had sufficient (non)immune-mediated disease to allow proper comparative analysis. In dataset A, 42% of the Risk-RA individuals developed arthritis during the two years follow up. In patients with unclassified joint swelling in dataset B and B 55 and 69% were diagnosed with an immune-mediated rheumatic disease after one year of follow-up. Table 1 describes the research individuals in more details.

**TABLE 1:** Patient characteristics and outcome in each dataset.

	Dataset A	Dataset B	Dataset C
N	50	51	73
Age (median, range)	48 (22–73)	54 (19–82)	59 (19–84)
Sex (% female)	44 (88)	36 (71)	37 (51)
Non-immune-mediated outcome*	29	23	22
Immune-mediated outcome*	21	28	51
Rheumatoid arthritis (%)	–	5 (10)	8 (11)
Undifferentiated arthritis (%)	–	4 (8)	10 (14)
RS3PE (%)	–		4 (5)
Reactive arthritis (%)	–		7 (10)
Psoriatic arthritis (%)	–	8 (16)	5 (7)
MCTD or vasculitis (%)	–	3 (6)	6 (8)
Sarcoid (%)	–	1 (2)	6 (8)
Paramalignant (%)	–		5 (7)
Systemic sclerosis (%)	–	1 (2)	
Spondyloarthropathy (other than reactive and psoriatic arthritis) (%)	–	4 (8)	
Systemic Lupus Erythematosus (%)	–	1 (2)	
Osteoarthritis (%)	–	22 (45)	17 (23)
Gout (%)	–	(2)	**5 (7)**

*The immune-mediated outcome was the development of inflammatory arthritis in dataset A and the development of an immune-mediated rheumatic disease in dataset B and C. RS3PE, remitting seronegative symmetrical synovitis with pitting edema. Bold values of the numbers is n (%).

### Discriminatory Ability of the Total Score

**FIGURE 1:**
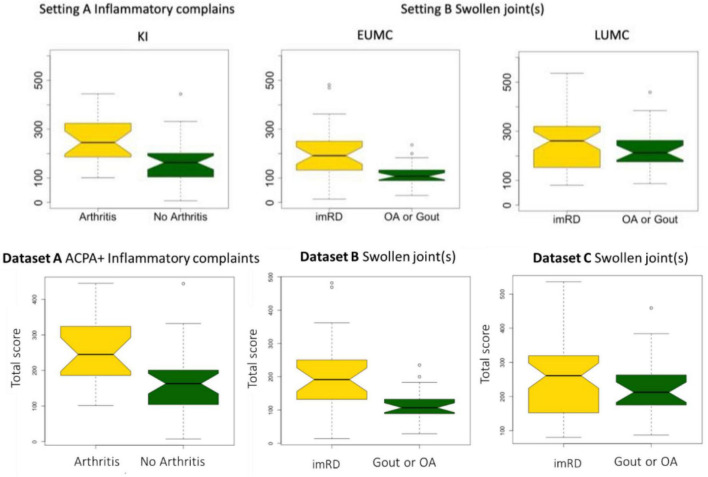
Total score in each dataset. Boxplot depicting the differences in total score between those who developed arthritis (dataset A) or an immune-mediated rheumatic disease (imRD) (datasets B and C) and those who did not.

In each cohort there was a wide variety in scores. Overall, patients who developed an immune-mediated disease after one or two years had a significantly higher *Rheumatic?* score at recruitment compared to those who did not: *P* < 0.0001 in all centers (Table 2 and Figure 1).

**TABLE 2:** Total *Rheumatic?* score in each dataset.

	Mean score	Median	Min	Max	*P*-value*
**Dataset A**					
All	203	186	7	445	
Arthritis	260	245	101	445	<0.0001
No arthritis	161	163	7	444	
**Dataset B**					
All	164	134	14	482	
Immune-mediated RD	204	191	14	482	<0.0001
Non-immune-mediated RD	115	107	29	235	
**Dataset C**					
All	240	234	80	536	
Immune-mediated RD	245	262	80	536	<0.0001
Non-immune-mediated RD	229	212	87	459	

*P-values are calculated using the Wilcoxon rank test. RD, rheumatic disease.

In dataset A, the individuals who did not develop arthritis had a lower median score at inclusion as compared with individuals who developed arthritis (163 vs. 245, *P* < 0.0001). Similarly, the lower scoring bound was substantially lower in those who did not develop arthritis (7 vs. 101). In dataset B, we observed a similar distinct difference in median score between the patients who developed a non-immune-mediated and immune-mediated rheumatic disease: 107 vs. 191, *P* < 0.0001. There was no clear difference in the lower bound of scores (14 and 14), but the upper bound differed greatly (235 vs. 482). In dataset C, all patients scored at least 80 points. Again, we found a difference in median score between those who developed an imRD and those who did not, but this difference was smaller than in the other centers (212 vs. 262, *P* < 0.0001). Here too, the maximum score differed between the two groups: 459 vs. 536.

**FIGURE 2:**
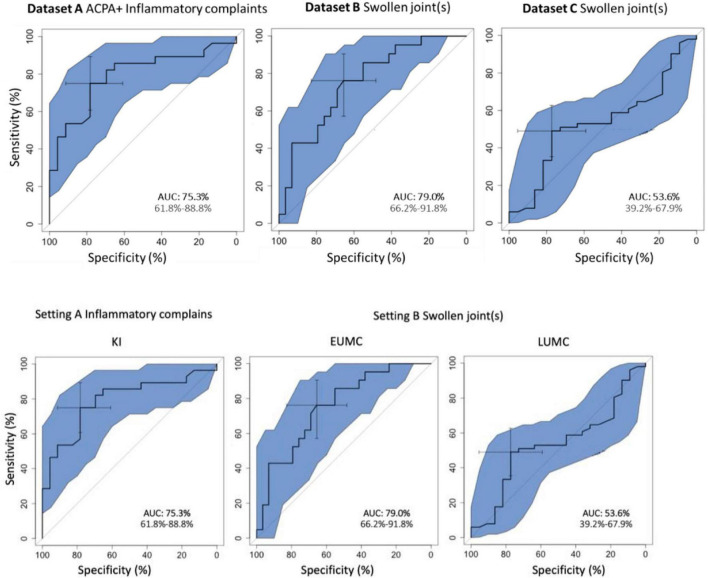
(Area under) the receiver operating curve for the total score of *Rheumatic?*

The overall discriminatory performance, as calculated with the AUC-ROC (95% Confidence Interval), was good in dataset A [75.3%, (61.8–88.8%)] and dataset B [79.0%, (66.2–91.8%)], but less so in dataset C [53.6%, (39.2–67.9%)] (Figure 2).

### Performance of *Rheumatic*? Thresholds

In dataset A, consisting of individuals presenting without arthritis but with ACPA positivity and musculoskeletal complaints, the individuals who developed arthritis passed threshold 1 (visit a general physician) substantially more often than those who did not develop arthritis (28 vs. 16%, Table 3). This resulted in a sensitivity and specificity of 67 and 72% for the recommendation to visit a primary care physician. For threshold 2 (visit a rheumatologist), only 1 of 21 patients passed the threshold.

**TABLE 3:** Performance of *Rheumatic?* in each cohorts in identifying immune-medicated rheumatic diseases using the predefined thresholds.

	Dataset A		Dataset B		Dataset C	
	Arthritis	No arthritis	imRD	No imRD	imRD	No imRD
Not passing threshold 1	7	21	11	20	17	5
Passing threshold 1	14	8	17	3	34	17
Sensitivity	67%		61% (41–78%)		67% (52–79%)	
Specificity	72%		87% (66–97%)		23% (8–45%)	
Not passing threshold 2	20	29	26	22	44	20
Passing threshold 2	1	0	2	1	7	2
Sensitivity	5%		7% (1–24%)		14% (6–25%)	
Specificity	100%		96% (78–100%)		91% (71–99%)	

*imRD, Immune-mediated rheumatic disease; RA, Rheumatoid arthritis. Between the brackets is the 95% Confidence Interval.*

In dataset B and C we tested the prediction of developing an immune mediated disease after 1 year in patients with unclassified arthritis. Patients who developed an immune-mediated rheumatic disease vs. those who did not, passed threshold 1 in 33 vs. 6% and 47 vs. 23% of times in dataset B and C, respectively. This resulted in a sensitivity of 0.61 and 0.67 and a specificity of 0.87 and 0.23 for passing threshold 1 in dataset B and C respectively. Threshold 2 was passed only in 4 vs. 2% and 10 vs. 3%, for immune-mediated vs. non-immune-mediated rheumatic disease in dataset B and C, respectively.

## Discussion

### Principal Results

We describe a pilot validation study of, to our knowledge, the first multilingual DDSS developed specifically for people suspecting an emerging or recent onset of immune mediated rheumatic disease. To our knowledge, this is also the first multicenter validation study of a rheumatic DDSS. In our pilot study we tested the performance of *Rheumatic?* in different settings using data from 175 individuals from three different European university centers. We observed a high performance of this online tool in differentiating individuals with emerging or existing immune-mediated rheumatic conditions vs. those without when using the total score. We tested *Rheumatic?* in the setting of individuals with various musculoskeletal complaints but without joint swelling, where only a subset developed an immune-mediated arthritis after an observation period of 2 years. Here the AUC-ROC was good. We furthermore tested the tool in the setting of patients with unclassified arthritis, including a subset of patients with gout or osteoarthritis (called non-.immune-mediated arthritis). The result was good in dataset B (AUC-ROC = 79%) and less good in dataset C (AUC-ROC = 54%).

The comparison between the data of the three different centers provides some interesting insights. We used data from existing cohorts which all had their own specific approaches for selecting participants. Consistent with the selection criteria (selection of patients without a suspicion of an immune mediated cause of joint swelling dataset A and B) the best discriminatory ability is seen in dataset A and B. The lack of reproducibility in dataset C and the much higher minimum scores there, could theoretically have two reasons (a) the tools is not discriminative in this population (b) in dataset C patient with gout and osteoarthritis have a similar inflammatory pattern of complaints as those with an immune mediated diseases. Likely, the latter is the case, Dataset C contains patients for whom the rheumatologist *a priori* considered an (auto)immune-mediated condition and thus will score high on the *Rheumatic?* questions addressing inflammatory pattern, making it harder to differentiate them from the patients with true immune mediated diseases. This is underlined by the overall higher scores of patients in this dataset.

*Rheumatic?* has expert-based thresholds for several rheumatic diseases, where passing threshold 1 and 2 suggest visiting a general doctor or rheumatologist, respectively. Here we observed that threshold 2 is highly specific for immune-mediated condition. Sensitivity, however, was unacceptably low as a results of identifying far too few patients with immune-mediated conditions. The sensitivity of threshold 1 was much better, though not optimal, with 0.61–0.67. The results of our pilot study suggest that the thresholds need to be optimized before being used in routine settings.

### Limitations

Our study has several limitations. Since this pilot study was the first quantitative study to the performance of *Rheumatic?* it lacked prior effect estimation to use for a power analysis. Our selected small number of patients was based on available resources and the selection focused on ensuring a sufficient prevalence of immune-mediated vs. non-immune-mediated rheumatic conditions. Hereby, the datasets do not reflect the true prevalences of the individual diseases. Thus we could not calculate the positive and negative predictive values nor optimize the threshold in the current data. However, the purpose of the current study was not to build prediction model, but to explore the possible discriminatory power of *Rhuematic?* in different clinical settings.

Furthermore, retrospectively entering symptoms (dataset B and C) described by patients might have introduced a DDSS usage bias. Also, some of the patients classified as not having an immune medicated condition in dataset A might still have developed such condition after longer follow-up. Our study does provide encouraging results which support future larger, prospective, studies. Linking genetic and immunological biomarkers, currently under development in our rheumatology units, to symptoms might optimize the tool performance in the future. Based on the current results, we recently initiated new studies, which will further help to improve *Rheumatic?* performance; a prospective multicenter study at the rheumatology outpatient and a population-wide study where people with musculoskeletal complaints are recruited online and followed for a year.

### Comparison With Prior Work

In 1991, Moens et al. concluded that rheumatology is a suitable domain for computer-assisted diagnosis (13). Despite the elapsed time, such systems are still not part of standard rheumatology care. Alder et al. concluded in a review in 2014 that the validation process of rheumatology DDSS was in general underappreciated and none of the systems seemed to have succeeded in daily practice (12). Patients and rheumatologists, however, still seem to believe in the positive potential impact of a patient facing DDSS (4, 25).

An RMD specific DDSS, based on a fuzzy cognitive map technique showed a diagnostic accuracy of 87% in a validation study with only 15 vignette cases (26). In a prospective pilot study, 34 patients completed an NHS and WebMD symptom checker. Only 4 out of 21 patients with immune-mediated arthritis were given a first diagnosis of rheumatoid arthritis or psoriatic arthritis (27). The first randomized controlled trial evaluating the performance of two SCs in rheumatology revealed very limited accuracy (Sensitivity: 54%, 54% Specificity 52%, 55%), despite both SCc already being used by thousands of patients (9). People suspecting axial spondyloarthritis (axSpA), using an online-self-referral were diagnosed in 19.4% with axSpA (14). This proportion being significantly higher than the assumed 5% prevalence of axial SpA in patients with chronic back pain (28).

Current research suggests that the diagnostic accuracy of DDSS are user dependent (29). The effectiveness of DDSS could also depend on the patient’s eHealth literacy (2).

A major strength of our study lies in its multicenter approach, the usage of true cases instead of patient vignettes and the relatively large validation sample size compared to previous studies (26, 27). The risk-adverse retrospective validation scenario was deliberately chosen. The majority of currently available symptom checkers seem to skip this validation process, providing little scientific evidence for their use. In these studies, the DDSS that were evaluated often use patient-vignettes instead of true data (17, 19). We believe that focusing on improving an international overarching DDSS could boost quality standards in rheumatology by increasing transparency, objectivity and decreasing redundant single-center efforts (15).

## Conclusion

By incorporating input from patients, rheumatologists and digital designers from multiple countries, a multilingual DDSS (*Rheumatic?*) for people suspecting a rheumatic disease was created. This first validation study shows that *Rheumatic?* is capable of differentiating immune-mediated rheumatic conditions from other musculoskeletal problems in the setting a) of patients who do not have arthritis and are considered to be at risk for RA and b) the group of patients with joint swelling. However, in the setting of arthritis where a clinician already suspected an immune mediated condition *Rheumatic?* was not capable of differentiation those who did and did not develop an immune mediated rheumatic disease. Future prospective, patient-lead, and independent studies are necessary to continuously validate and improve the performance of Rheumatic?

## Data Availability Statement

Study data (scores and diagnoses) can be obtained upon request to the data-owning authors.

## Ethics Statement

Ethical approval was obtained from the local ethical committees. Patients provided written informed consent when entering a cohort.

## Author Contributions

RK and TM ran the statistical analyses. LK and AnC initiated the development of the tool. SS, AL, TB, SE, LK, AnC, MJ, RK, and JK built and improved the tool. RK, JK, AH, AC, DS, AK, GS, and TH collected the data. TB, SE, MJ, SS, AL, LK, AC, AH, LB-V, and MM developed the multilanguage Rheumatic? tool. RK and JK drafted the first version of the manuscript. As employees of Elsa Science, TB, SE, SS, and AL did not comment on the analytical approach. All authors gave final approval of the version to be published and were involved in the interpretation of the data and drafting of the manuscript.

## Conflict of Interest

TB, SE, SS, and AL were employees of the companies that developed the *Rheumatic*? tool. LK is one of the founders of the Elsa Science company, but has no financial interests in or compensations from the company. The remaining authors declare that the research was conducted in the absence of any commercial or financial relationships that could be construed as a potential conflict of interest.

## Publisher’s Note

All claims expressed in this article are solely those of the authors and do not necessarily represent those of their affiliated organizations, or those of the publisher, the editors and the reviewers. Any product that may be evaluated in this article, or claim that may be made by its manufacturer, is not guaranteed or endorsed by the publisher.
